# Protonation of Piezo1 Impairs Cell-Matrix Interactions of Pancreatic Stellate Cells

**DOI:** 10.3389/fphys.2020.00089

**Published:** 2020-02-14

**Authors:** Anna Kuntze, Ole Goetsch, Benedikt Fels, Karolina Najder, Andreas Unger, Marianne Wilhelmi, Sarah Sargin, Sandra Schimmelpfennig, Ilka Neumann, Albrecht Schwab, Zoltan Pethő

**Affiliations:** ^1^Institute of Physiology II, University of Münster, Münster, Germany; ^2^Institute of Physiology, University of Lübeck, Lübeck, Germany

**Keywords:** piezo1, pancreatic stellate cells, pancreatic cancer, fibrosis, pH homeostasis, cell migration

## Abstract

Pancreatic ductal adenocarcinoma (PDAC) is characterized by an acidic and fibrotic stroma. The extracellular matrix (ECM) causing the fibrosis is primarily formed by pancreatic stellate cells (PSCs). The effects of the altered biomechanics and pH landscape in the pathogenesis of PDAC, however, are poorly understood. Mechanotransduction in cells has been linked to the function of mechanosensitive ion channels such as Piezo1. Here, we tested whether this channel plays crucial roles in transducing mechanical signals in the acidic PDAC microenvironment. We performed immunofluorescence, Ca^2+^ influx and intracellular pH measurements in PSCs and complemented them by live-cell imaging migration experiments in order to assess the function of Piezo1 channels in PSCs. We evaluated whether Piezo1 responds to changes of extracellular and/or intracellular pH in the pathophysiological range (pH 6.6 and pH 6.9, respectively). We validated our results using Piezo1-transfected HEK293 cells as a model system. Indeed, acidification of the intracellular space severely inhibits Piezo1-mediated Ca^2+^ influx into PSCs. In addition, stimulation of Piezo1 channels with its activator Yoda1 accelerates migration of PSCs on a two-dimensional ECM as well as in a 3D setting. Furthermore, Yoda1-activated PSCs transmit more force to the surrounding ECM under physiological pH, as revealed by measuring the dislocation of microbeads embedded in the surrounding matrix. This is paralleled by an enhanced phosphorylation of myosin light chain isoform 9 after Piezo1 stimulation. Intriguingly, upon acidification, Piezo1 activation leads to the initiation of cell death and disruption of PSC spheroids. In summary, stimulating Piezo1 activates PSCs by inducing Ca^2+^ influx which in turn alters the cytoskeletal architecture. This results in increased cellular motility and ECM traction, which can be useful for the cells to invade the surroundings and to detach from the tissue. However, in the presence of an acidic extracellular pH, although net Ca^2+^ influx is reduced, Piezo1 activation leads to severe cell stress also limiting cellular viability. In conclusion, our results indicate a strong interdependence between environmental pH, the mechanical output of PSCs and stromal mechanics, which promotes early local invasion of PDAC cells.

## Introduction

Pancreatic ductal adenocarcinoma (PDAC) is the most common primary malignant tumor of the exocrine pancreas, and credits for an abysmal prognosis among all forms of cancer. PDAC is characterized by a collagen-rich desmoplastic stroma that compresses the tumor, which thereby reaches hydrostatic pressure levels of up to 100 mmHg (DuFort et al., 2016). In addition, the desmoplastic PDAC microenvironment is markedly acidic reaching even below pH 6.5 (Cruz-Monserrate et al., 2014; Pedersen et al., 2017). Over time the acidic extracellular pH (pH_e_) can also lead to an acidification of the tightly regulated intracellular pH (pH_i_) (Riemann et al., 2013). Consequently cellular migration is affected and cell death pathways are initiated in most eukaryotic cells (Martin et al., 2011; Justus et al., 2013; Schwab and Stock, 2014).

In PDAC, the desmoplastic stroma is mainly produced by pancreatic stellate cells (PSCs) (Xue et al., 2018). Activated PSCs have a myofibroblast-like phenotype with expression of markers such as α-smooth muscle actin (αSMA). They are involved in fibrotic remodeling of the extracellular matrix (ECM). In PDAC this leads to the formation of a characteristic tumor niche that insulates tumor cells from the immune system and from the effect of chemotherapeutic drugs (Lonardo et al., 2012). Moreover, PSCs invade the surrounding tissues together with PDAC cells, thus actively facilitating PDAC metastasis (Liu et al., 2019).

Although not studied as extensively as in PDAC cells, it has become clear that Ca^2+^ signaling plays an important role in PSC physiology and pathophysiology (Ferdek and Jakubowska, 2017; Storck et al., 2017). Ca^2+^ signals can be elicited through multiple mediators, e.g., bradykinin, angiotensin, ATP as well as environmental pressure (Hennigs et al., 2011; Won et al., 2011; Fels et al., 2016). Ion channels that have been studied to mediate Ca^2+^ influx in PSCs are manifold, ranging from CRAC channel, P2X_7_ receptor to a wide array of TRP channels (TRPV4, TRPM7, TRPC1, TRPC3, TRPC6) (Haanes et al., 2012; Fels et al., 2016; Jakubowska et al., 2016; Nielsen et al., 2017; Storck et al., 2017).

Recent evidence implies that activation of Piezo1—a mechanosensitive cation channel that mediates preferentially Ca^2+^ influx upon mechanical stress—is essential for the formation of the fibrotic pancreatic stroma in chronic pancreatitis (Romac et al., 2018). In concordance with this finding, our previous work shows that PSCs express a large amount of Piezo1 mRNA (Fels et al., 2016). Activation of Piezo1 in PSCs would lead to Ca^2+^ influx which in turn prompts a myriad of cellular responses in cancer (Fels et al., 2018; Pethő et al., 2019). These responses include phosphorylation of regulatory myosin light chains (MYLs) and activation of calmodulin-dependent cellular pathways that eventually coordinate gene expression, secretion of extracellular matrix, cellular contractility and migration, proliferation and cell death (Tsai et al., 2015; Gryshchenko et al., 2016).

Ca^2+^ influx will also lead to the downstream activation of calcium-activated K^+^ channels (e.g., K_Ca_3.1) which in turn feeds back on Piezo1 channels by their impact on the cell membrane potential (Storck et al., 2017; Pethő et al., 2019). In PDAC the characteristic mechanical properties of the desmoplastic microenvironment are expected to lead to continuous Piezo1 activation and hence, to sustained Ca^2+^ influx. The Ca^2+^ signals could be interpreted by the cells as a stiffness-derived survival signal in case of moderate, controlled Ca^2+^ influx. Another possibility could be that the cells get overloaded by an excessive or prolonged Ca^2+^ signal and enter cell death pathways (Harr and Distelhorst, 2010).

The open probability of Piezo1 can be allosterically enhanced by the small-molecular agonist Yoda1 (Syeda et al., 2015), which is known to induce Ca^2+^ influx in transformed fibroblasts and myofibroblasts (Blythe et al., 2019; Chubinskiy-Nadezhdin et al., 2019). In contrast, Piezo1 can be inhibited by rendering it in an inactive conformation by extracellular acidification below pH 6.9, as demonstrated by a thorough patch clamp study (Bae et al., 2015). While Piezo1 is inherently mechanosensitive even in artificial lipid bilayers (Syeda et al., 2016), it is widely accepted that channel function is fine-tuned in live cells by intracellular tethers such as the actin-myosin cytoskeleton as well as by extracellular tethers such as the ECM (Ranade et al., 2015; Nourse and Pathak, 2017).

We hypothesize that Piezo1-mediated mechanosensation in PSCs is heavily dependent on the pH microenvironment and is fine-tuned by its interaction with intra- and extracellular tethers. In this study, we tested, whether Piezo1 is still active in the presence of an acidic pH_i_ and/or pH_e_, conditions that resemble the acidic tumor core. As validation, we inspected the pH-dependence of Piezo1-mediated Ca^2+^ influx in Piezo1-transfected HEK293 cells. Lastly, we investigated Piezo1-cytoskeleton and Piezo1-ECM interactions in PSCs in 2D and 3D systems and whether they are impacted by environmental pH.

## Materials and Methods

### Animal Experiments

Animal experiments were carried out with the approval of the local ethics committee for animal care (*Landesamt für Natur, Umwelt und Verbraucherschutz Nordrhein-Westfalen*, permit number 84-02.05.50.15.010).

### Murine PSC Isolation and Culture

PSCs were isolated from healthy wild-type C57BL/6J mice aged 8–12 weeks as described previously (Fels et al., 2016). Briefly, murine pancreata were isolated, and then enzymatically digested with 0.1% collagenase P (Sigma-Aldrich, Merck KGaA, Darmstadt, Germany) for 25 min at 37°C. After centrifugation, the homogenized tissue was resuspended in cell culture medium (DMEM/Ham F12 1:1, supplemented with 10% FCS and 1% penicillin/streptomycin) and seeded onto FCS-coated tissue culture dishes for 2 h. Afterwards, non-adherent cells were forcefully washed off the tissue culture plate, resulting in a homogeneous PSC culture. PSCs were used for experiments after two passages.

### Plasmids and Transfection

The pRK9 plasmid containing Piezo1-IRES-GFP (size 13,701 bp) was kindly provided by Prof. Gary R. Lewin's laboratory. The IRES promoter allows GFP to be transcribed only when Piezo1 has been transcribed, too. To create a suitable control plasmid, we removed the Piezo1 and IRES coding DNA from the plasmid. For this, we applied the restriction enzymes NotI and MscI (New England Biolabs, Ipswitch, MA, USA) to the Piezo1-IRES-GFP plasmid for 1 h at 37°C according to the manufacturer's instructions. Thus, the resulting plasmid was a structurally analogous control plasmid (size 5,411 bp) coding GFP but not the Piezo1 or IRES. Plasmid fragments were separated by gel electrophoresis on 1% agarose gel, then excised and purified using the QIAquick Gel Extraction Kit (Qiagen, Hilden, Germany). Following purification, DNA ends were blunted using the Klenow DNA Polymerase I, and finally, the 3′ and 5′ ends were ligated using T4 DNA ligase following the manufacturer's manual (New England Biolabs, Ipswitch, MA, USA).

A total of 25,000 HEK293 cells were seeded in glass-bottom dishes (Cell E&G, San Diego, USA) and after overnight incubation in 10% FCS-supplemented DMEM at 37°C, cells were transiently transfected with either the pRK9 Piezo1-IRES-GFP (HEK^Piezo1+^) or the pRK9-GFP control plasmid (HEK^GFP+^) using 1 μg plasmid DNA and 40 μl Lipofectamine® 3000 (Sigma-Aldrich, Merck KGaA, Darmstadt, Germany) in Opti-MEM reduced serum media (Thermo Fisher Scientific, Inc., Waltham, MA, USA). Twenty-four to forty-eight hours after transfection, HEK^Piezo1+^ and HEK^GFP+^ cells could be identified in the GFP channel of the fluorescence microscope (Visitron Systems, Puhheim, Germany) and subsequently used for Mn^2+^ quench experiments.

### Immunocytochemistry

For immunocytochemistry, 30,000 PSCs were plated onto collagen I (Corning, New York, NY, USA) coated glass coverslips and cultured for 48 h. Following incubation, PSCs were treated with 5 μM Yoda1 (Tocris Bioscience, Bristol, UK) or solvent (0.2% DMSO) for 1 h. To monitor the effects of blebbistatin, cells were additionally treated with 5 μM blebbistatin (Sigma-Aldrich, Merck KGaA, Darmstadt, Germany) for 15 min.

For visualizing Piezo1 membrane expression, live cell staining was performed as described previously by our group (Waschk et al., 2011; Storck et al., 2017). First, cells were blocked with Ringer's solution (122.5 mM NaCl, 5.4 mM KCl, 1.2 mM CaCl_2_, 0.8 mM MgCl_2_, 5.5 mM d-glucose and 10.0 mM HEPES, titrated to pH 7.4) supplemented with 1% bovine serum albumin (Sigma-Aldrich, Merck KGaA, Darmstadt, Germany) for 1 h on ice. This was followed by staining with the primary antibody against mPiezo1 (Proteintech, Manchester, UK, #15939-1-AP, 1:200) for 2 h at 4°C. This antibody recognizes an extracellular epitope of the channel protein so that cell permeabilization was not necessary. After washing five times, coverslips were fixed in 3.5% paraformaldehyde for 20 min at 4°C. Afterwards, Alexa 488-conjugated secondary antibody (Invitrogen, Carlsbad, CA, USA, 1:500) was applied for 1 h at room temperature. Finally, after washing 3 times with PBS, 0.1% DAPI (Sigma-Aldrich, Merck KGaA, Darmstadt, Germany) was applied in DAKO mounting solution (DAKO Deutschland GmbH, Hamburg, Germany) and coverslips were mounted onto slides.

For other immunofluorescence assays, fixation was performed on ice using 3.5% paraformaldehyde for 1 h, followed by washing with PBS 3 times. Cells were subsequently permeabilized using 0.25% Triton-X-100 in PBS, then blocked using 10% goat serum for 1 h at room temperature. Primary antibodies against αSMA (Sigma-Aldrich, Merck KGaA, Darmstadt, Germany, #A2547, 1:200), phosphorylated myosin light chain 9 (P-MYL-9; Invitrogen, Carlsbad, CA, USA, 1:100), and non-muscle myosin IIa (NMIIA) (Novus Biologicals, Littleton, CO, USA, 1:100) were applied overnight at 4°C. After washing three times with PBS, Alexa 488-conjugated secondary antibodies against mouse (Invitrogen, Carlsbad, CA, USA, 1:500) and rabbit (Invitrogen, Carlsbad, CA, USA, 1:500) were applied for 1 h at room temperature. Lastly, after washing three times with PBS, 0.1% DAPI was applied in DAKO mounting solution and coverslips were mounted onto slides.

**Figure 1:**
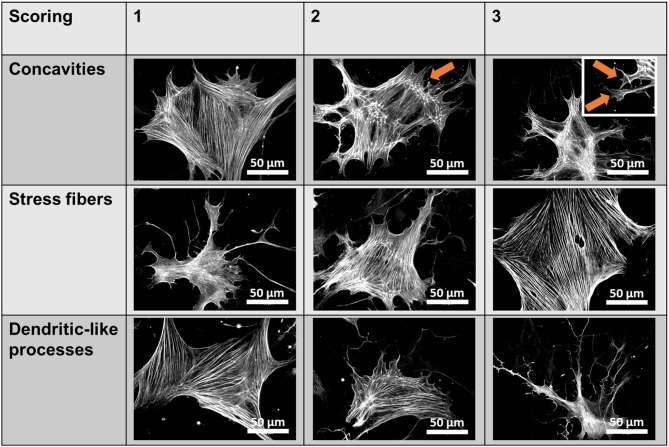
Blebbistatin scoring system. Scores ranging from 1 to 3 were given for individual pancreatic stellate cells (PSCs) based on three criteria (membrane concavities, stress fibers, and dendritic-like processes), which are characteristic morphological alterations observed in blebbistatin-treated PSCs. The representative images show αSMA immunocytochemistry of PSCs. The membrane concavities are highlighted with orange arrows.

Fluorescent images were acquired using a Zeiss Axiovert 200 inverted fluorescence microscope (Zeiss, Oberkochen, Germany) at 40× or 100× magnification. Quantification of Piezo1 channel density was performed manually by counting bright spots representing channels in 10 rectangular regions of 7.6 μm × 7.6 μm per cell and subtracting the mean number of spots in equivalent extracellular regions. For P-MYL9 and NMIIA immunocytochemistry fluorescence ratios of P-MYL9/NMIIA were assessed in an unpaired manner, as both primary antibodies originate from the same species (rabbit). First, the total cellular P-MYL9 and NMIIA intensities of 30 cells were measured using similar exposure and illumination settings. Afterwards, the amount of P-MYL9 was normalized to the total NMIIA of a randomly assigned cell. To evaluate the effects of blebbistatin, a scoring system was used to measure the stress fibers, the dendrite-like processes and cellular concavities (Figure 1).

### Western Blot

Western blots of Piezo1, GAPDH, MYL2, and phospho-MYL2 (P-MYL2) were performed as described previously (Bulk et al., 2017). Total protein of PSCs was extracted using radioimmunoprecipitation assay (RIPA) buffer [50 mmol/l Tris, 150 mmol/l NaCl, 0.1% SDS, 0.5% sodium deoxycholate, 1% NP-40, and 1% Complete Mini protease inhibitor (Roche, Mannheim, Germany)]. Protein concentration of the samples was determined with Pierce™ BCA Protein Assay Kit (Thermo Fisher Scientific, Inc., Waltham, MA, USA). Fifteen microgram of denatured total cellular protein was applied to each lane of a 15% polyacrylamide gel for electrophoresis at 80 mV. After overnight transfer to PVDF membranes at 4°C, we blocked the membrane with PBS containing 5% skim milk for 1 h, then incubated the blots with primary antibodies against mPiezo1 (Proteintech, Manchester, UK, #15939-1-AP, 1:100), P-MYL2 (1:500, MYL-Ser19 mouse mAb, #3675 Cell Signaling Technology, Danvers, Ma, USA), or MYL2 (1:500, MYL2 rabbit Ab, #3672 Cell Signaling Technology, Danvers, Ma, USA), and for housekeeping control GAPDH (1:2,000, GAPDH mouse mAb, #ab125247, Abcam, Cambridge, UK) overnight at 4°C. After washing three times with PBS, we applied HRP-conjugated goat-anti rabbit (1:10,000, Goat Anti-Rabbit IgG H&L, #ab6721, Abcam, Cambridge, UK) in case of Piezo1 and P-MYL2; and goat anti mouse secondary antibody (1:10,000, Goat Anti-Mouse IgG H&L, #ab6708, Abcam, Cambridge, UK) in case of MYL2. Chemiluminescence was detected using a commercial detection system (Chemidoc MP, Bio-Rad, Hercules, CA, USA), and band intensities were evaluated with ImageJ.

### Calcium Influx Measurement

We applied the Mn^2+^ quench technique to monitor calcium influx into the cells (Merritt et al., 1989; Fels et al., 2016; Nielsen et al., 2017). Mn^2+^ largely mimics Ca^2+^ as it enters cells via similar pathways. In contrast to Ca^2+^ ions, Mn^2+^ ions quench the fluorescence emission of the calcium sensitive dye Fura-2. These experiments are performed at the Ca^2+^ insensitive, isosbestic excitation wavelength of Fura-2. The Mn^2+^-induced drop of the Fura-2 fluorescence is largely proportional to the transmembrane influx of Ca^2+^.

**Figure 2:**
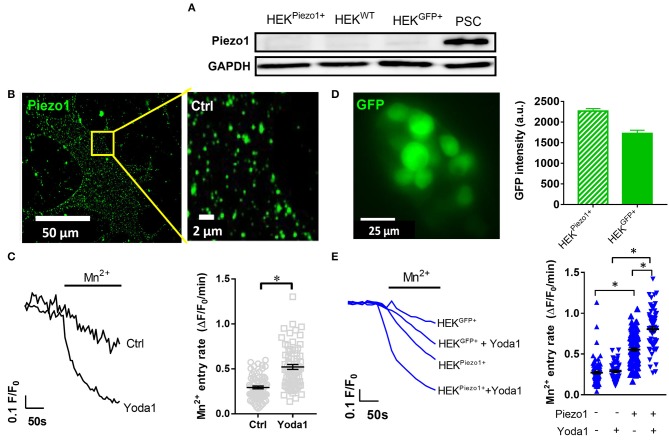
Piezo1 is functionally expressed in PCSs. **(A)** Representative Western blots of Piezo1 compared to GAPDH in PSCs, wild-type HEK293 cells (HEK^WT^) and HEK293 cells transfected with control plasmid (HEK^GFP+^) or Piezo1 (HEK^Piezo1+^) (*N* = 3). **(B)** Piezo1 immunocytochemistry of non-permeabilized PSCs shows homogeneous channel distribution in the cell membrane. **(C)** Representative Mn^2+^-induced Fura-2 quench traces of PSCs in the presence of 20 μM Yoda1 or equivalent amount of the solvent, 0.075% DMSO (Ctrl). The scatter plots on the right show the respective values of Mn^2+^ quench slopes, the Mn^2+^ entry rate. The black lines indicate mean ± SEM. (*n* = 72, *N* = 3 for each condition). **(D)** Representative GFP image of Piezo1-transfected HEK293 cells. Only GFP^+^ cells were evaluated in the Mn^2+^ quench experiments. The bar chart on the right shows the evaluation of GFP signal intensities after control plasmid (HEK^GFP+^) and Piezo1 (HEK^Piezo1+^) transfection compared to untransfected HEK293 cells with intensities <100. **(E)** HEK293 cell derived Mn^2+^ quench traces in the presence of the solvent 0.025% DMSO or 5 μM Yoda1 in case of HEK^GFP+^ (*n* = 82; *n* = 50, respectively, *N* = 3) or HEK^Piezo1+^ cells (*n* = 106; *n* = 60, respectively, *N* = 3). The scatter plots indicate Mn^2+^ entry rates of individual cells for each condition. The black lines indicate mean ± SEM. *p < 0.05.

After staining the cells with the Ca^2+^-sensing dye Fura-2-AM (#F1221, Thermo Fisher Scientific, Inc., Waltham, MA, USA; 6 μM) for 20 min at 37°C in HEPES buffered Ringer's solution, cells were visualized using a ionic imaging setup consisting of a fluorescence microscope, a high-speed shutter and a polychromator (Visitron Systems, Puchheim, Germany). The proper isosbestic excitation wavelength, at which the emission is Ca^2+^ independent, was determined using the Ca^2+^ ionophore ionomycin (not shown). Thereafter, measurements were conducted at an excitation wavelength of 357 nm and fluorescence emission was recorded at 510 nm. Images were acquired in 10 s intervals. The experimental protocol started with an initial 3 min incubation in Ringer's solution. This was followed by another 3 min incubation in a Ca^2+^-containing, Mn^2+^ supplemented Ringer's solution for the experiments depicted in Figures 2C, **5A**. For all other measurements we used a Ca^2+^-free, Mn^2+^ supplemented Ringer's solution (Mn^2+^ Ringer's). The Mn^2+^ concentration in Mn^2+^ Ringer's was 400 μM in case of PSCs and 1 mM in case of HEK293 cells. Also, in HEK293 cells, GFP caused negligible interference with the Fura-2 signals (not shown).

Data analysis was performed by measuring fluorescence intensities over the whole cell area and correcting it for background fluorescence. The extracted fluorescence intensities F were normalized to the initial fluorescence intensities F_0_ determined under control conditions in the presence of Ringer's solution (F/F_0_). For each cell, slopes of linear regression (ΔF/F_0_/min) were calculated before and after Mn^2+^ application during intervals of 30 s. Subsequently, the slope after Mn^2+^ application was subtracted by the slope in the presence of Ringer's solution to correct for potential photobleaching. Lastly, for easier interpretation, the inverse value of the Mn^2+^ quench was determined which directly correlates with Ca^2+^ influx.

### Determination of the Intracellular pH

We assessed the intracellular pH (pH_i_) of HEK293 cells using the fluorescent pH indicator BCECF-AM. Prior to the experiment, ${\text{HCO}}_{3}^{-}$-buffered cell culture medium was exchanged for the HEPES-buffered Ringer's solution for 15 min to equilibrate pH_i_ at 37°C. BCECF staining was performed using 1 μM BCECF-AM for 3 min in Ringer's solution (pH 7.4) at 37°C. Afterwards, cells were washed and continuously superfused with Ringer's solution for 3 min, followed by 3 min superfusion of acidified Ringer's solution (pH 6.6) or Ringer's solution supplemented with 30 mM sodium propionate (Sigma-Aldrich, Merck KGaA, Darmstadt, Germany). To maintain osmolarity the latter replaced 30 mM NaCl. Lastly, for calibration purposes, cells were permeabilized for protons using 1 μM nigericin (Sigma-Aldrich, Merck KGaA, Darmstadt, Germany) and superfused with a modified Ringer's solution (KCl 125 mM, MgCl_2_ 1 mM, CaCl_2_ 1 mM, and HEPES 20 mM) titrated to pH 7.5 and pH 6.5. Data were acquired with the same ionic imaging setup used for Ca^2+^ influx measurements measuring with dual excitation wavelengths of 440 and 490 nm and emission wavelength of 510 nm.

During data analysis, fluorescence intensities were measured over the whole cell area and corrected for background fluorescence. Subsequently, the 440 nm_ex_/490 nm_ex_ fluorescence intensity ratios were calculated. As BCECF fluorescence ratio follows a linear trend between pH 6.5 and pH 7.5, two-point calibration was performed using linear regression.

### Cell Migration and Spheroid Traction Experiments

Migration of PSCs was monitored using time-lapse video microscopy as described previously (Schwab et al., 2005; Fels et al., 2016). PSCs in cell culture medium were seeded in pre-coated 12.5 cm^2^ dishes, Here, the coating matrix mimics the desmoplastic stroma and contained the following: 40 μg/ml laminin (Sigma-Aldrich, Merck KGaA, Darmstadt, Germany), 40 μg/ml fibronectin (Sigma-Aldrich, Merck KGaA, Darmstadt, Germany), 800 μg/ml collagen I (Corning, New York, NY, USA), 12 μg/ml collagen III (Corning, New York, NY, USA), and 5.4 μg/ml collagen IV (BD Biosciences, Heidelberg, Germany). Cell migration was recorded in temperature-controlled chambers (37°C) with CMOS cameras for 12 h at 5 min intervals using the MicroCamLab 3.1 software (Bresser, Rhede, Germany). Quantitative analysis of single cell migration was based on the segmentation of PSC contours using the Amira software suite (Thermo Fisher Scientific, Inc., Waltham, MA, USA). From the segmented cell contours, cell velocity (μm/min) and translocation were calculated as described before (Dieterich et al., 2008). Migration velocity was defined as the displacement of the cell centroid as a function of time. Mean translocation represents the net distance covered during the entire experiment.

For spheroid formation, 5,000 PSCs were inserted into a hanging drop of 40 μL containing 0.31% methylcellulose (Sigma-Aldrich, Merck KGaA, Darmstadt, Germany) in cell culture medium. Spheroids formed spontaneously within 48 h. They were transferred into a modified matrix suitable for spheroid embedding containing 75 μg/ml laminin, 75 μg/ml fibronectin, 4.3 mg/ml collagen I, 27 μg/ml collagen III, and 12 μg/ml collagen IV. The matrix also contained 1 × 1,010 microbeads per ml (FluoSpheres 1 μm, Invitrogen, Carlsbad, CA, USA) to monitor matrix traction. Eighty microliter of matrix containing a spheroid was allowed to polymerize in 12.5 cm^2^ tissue culture dishes for 2 h at 37°C in 5% CO_2_, and subsequently 3 mL cell culture medium was added to the dishes supplemented with 50 ng/ml platelet-derived growth factor (PDGF). The experiments started after an equilibration period of another 2 h at 37°C in a 5% CO_2_/air atmosphere.

The fate of PSC spheroids in the three-dimensional matrix was monitored using time-lapse video microscopy for a total of 48 h, similarly to the single cell migration assay described above. As a readout of spheroid invasion, the equatorial cross-sectional area of a given spheroid after 24 h was divided by the initial spheroid area at *t* = 0 h. The number of cells detached from the spheroid after 24 h was counted. Spheroids apply a remarkable amount of traction toward the matrix, as visible in Video 1. The traction of the matrix was evaluated by quantifying the movement of 10 beads within a maximal radius of 500 μm neighboring the spheroid during a 12 h period. The mean velocity of the beads (μm/h) is a surrogate of the traction of the spheroid toward the surrounding matrix.

### Spheroid Histology and Confocal Reflectance Imaging

PSC spheroids were incubated for 24 h at pH 7.4 and pH 6.6 in the presence of 20 μM Yoda1 or vehicle (0.1% DMSO), respectively. Afterwards, the collagen matrix containing spheroids was scraped off the dishes using a scalpel. Spheroids in the collagen matrix were fixed in 4% paraformaldehyde, 15% saturated picric acid in 100 mM phosphate buffer overnight at 4°C, dehydrated via ascending ethanol series, and embedded in paraffin. For histology, thin sections (5–7 μm) were cut with a rotary microtome (Leica RM 2135), rehydrated, and a routine hematoxylin and eosin stain (HE) was performed. The hematoxylin stains cell nuclei in a blue color, whereas eosin stains the ECM as well as the cytoplasm pink.

To evaluate the consistence of PSC spheroids, first, total cross-sectional area of spheroids was measured in multiple sections of each spheroid. Afterwards, by thresholding according to Li (Li and Tam, 1998), the acellular area within each spheroid could be measured in ImageJ. Spheroid fragmentation was calculated by dividing the acellular spheroid area by total spheroid area.

### Assessment of Cellular Viability

MTT assays were performed with matrix-embedded PSC spheroids cultured at pH 7.4 and pH 6.6 with 20 μM Yoda1 or vehicle (0.1% DMSO), respectively, for 24 h. First, spheroids were transferred to a 96-well plate, medium was exchanged for one containing 0.5 mg/ml MTT reagent (Sigma-Aldrich, Merck KGaA, Darmstadt, Germany). Spheroids were incubated at 37°C in 5% CO_2_ for 2 h. Then medium was discarded and 100 μL DMSO were added in order to dissolve formazan crystals. Absorbance was measured at 546 nm and corrected for absorbance at 650 nm using a multi-well spectrophotometer (Beckman Coulter, Brea, CA, USA). MTT reduction was normalized to absorbance of spheroids cultured at pH 7.4 with vehicle.

Annexin V staining was performed with trypsin-digested PSC spheroids according to manufacturer's instructions. Briefly, PSC spheroids were extracted from the collagen gels manually, washed with PBS and then inserted into tubes containing 0.25% Trypsin-EDTA solution (Sigma-Aldrich, Merck KGaA, Darmstadt, Germany) and shaken at 250 rpm at 37°C for 10 min. After centrifugation at 200 g for 5 min, cells were resuspended in Annexin V binding buffer (Mybiosource, San Diego, CA, USA) and stained with Annexin V conjugated with Alexa 555 (A35108, Invitrogen, Carlsbad, CA, USA, 1:20) at RT for 15 min. Lastly, cells were washed with Annexin V binding buffer and visualized using a fluorescence microscope at 10× magnification in the red fluorescence channel and DIC (Zeiss Axio Observer, Zeiss, Oberkochen, Germany). Annexin V positivity was evaluated by manual thresholding in ImageJ for 3 visual fields per spheroid. Annexin V positive cells were counted and summed for all visual fields and divided by the total number of cells counted in the respective DIC images.

### Statistics

Data are presented as mean values ± S.E.M and following test for normality unpaired Student's *t*-tests or one-way ANOVA were performed with Tukey's *post-hoc* test where applicable using Graphpad Prism 7. Statistical significance was assumed when *p* < 0.05.

## Results

### Piezo1 Channels Are Expressed in Pancreatic Stellate Cells

We performed Western blot to investigate Piezo1 expression in whole-cell lysates of cell populations investigated in our study: namely in untransfected HEK293 cells, in HEK293 cells transiently transfected with control plasmid or Piezo1 and in PSCs (Figure 2A). We found that Piezo1 is abundantly expressed in PSCs, whereas overall Piezo1 was scarcely expressed in both untransfected and transiently transfected HEK293 cells. To visualize membrane expression of Piezo1 in PSCs, we labeled the channel using immunocytochemistry (Figure 2B). Piezo1 is expressed homogeneously in the cell membrane at a density of 0.52 ± 0.08 channels/μm^2^ (*n* = 300 regions of 30 cells, *N* = 3 experiments). Moreover, to assess functionality of the channels, we measured Piezo1-mediated Ca^2+^ influx using the Mn^2+^ quench technique in the presence or absence of the Piezo1 agonist, Yoda1. Here, we found that 20 μM Yoda1 almost doubles Ca^2+^ influx into PSCs, from 0.29 ± 0.02 to 0.52 ± 0.03 ΔF/F_0_/min (*p* < 0.0001, *N* = 3 experiments with *n* = 72 PSCs) (Figure 2C).

To validate the specificity of the Yoda1-induced Mn^2+^ quench assay, we compared Ca^2+^ influx into untransfected HEK293 cells with that into HEK293 cells transfected with a control plasmid or with Piezo1 (Figure 2E). As overall Piezo1 expression in the transiently transfected HEK293 cell population is low, we only measured Mn^2+^ quench in transfected GFP^+^ HEK293 cells (Figure 2D). GFP intensities of GFP^+^ control (1,724 ± 79, *n* = 523) and Piezo1 transfection (2,266 ± 61, *n* = 816) were proportionately increased compared to wild-type HEK293 autofluorescence with intensities < 100 (Figure 2D). Untransfected and control transfected HEK293 cells exhibit the same (*p* = 0.97) Yoda1-induced Mn^2+^ quench rate: 0.27 ± 0.02 ΔF/F_0_/min (*n* = 50, *N* = 3) and 0.28 ± 0.02 ΔF/F_0_/min (*n* = 82, *N* = 3), respectively (Figure 2E). In contrast, Piezo1-transfection leads to a 2-fold higher Mn^2+^ quench rate in HEK293 cells (0.55 ± 0.02 ΔF/F_0_/min, *n* = 106, *N* = 3). Interestingly and in line with the Western blot results shown in Figure 2A, we needed a considerably higher Mn^2+^ concentration for HEK293 cells than for PSCs to elicit comparable quench rates. Piezo1-mediated Ca^2+^ influx is distinctly amplified using 5 μM Yoda1 (0.81 ± 0.03 ΔF/F_0_/min, *n* = 60, *N* = 3) compared to solvent (0.025% DMSO) superfusion (Figure 2E). Collectively, these results provide evidence that PSCs express Piezo1 channels and that their function can be assessed with the Mn^2+^ quench technique.

### Piezo1 Channels Facilitate Pancreatic Stellate Cell Migration and Cell-ECM Interaction

**Figure 3:**
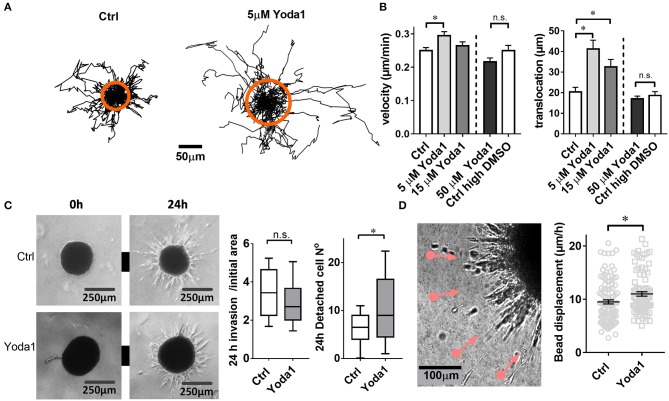
Yoda1-elicited responses in PSCs. **(A)** Trajectories of individual PSCs migrating on a 2D substrate in the absence or presence of 5 μM Yoda1. Trajectories are normalized to common starting points. The radii of the orange circles show the mean translocation in both cases. **(B)** Mean velocity and translocation, respectively, were calculated from the trajectories for a 6-h time span. Five micrometer and 15 μM Yoda1 treatments were compared to 0.075% DMSO (Ctrl), whereas 50 μM Yoda was compared to 0.25% DMSO (Ctrl high DMSO). **(C)** Phase contrast images of spheroids at time points 0 and 24 h show matrix invasion of the vehicle-treated (Ctrl) and 20 μM Yoda1-treated spheroids. The left plot shows spheroid area after 24 h normalized to the initial area at 0 h (Ctrl: *n* = 54, *N* = 8; Yoda1 *n* = 51, *N* = 8). The right plot indicates the number of cells detached from the spheroids (Ctrl: *n* = 45, *N* = 8; Yoda1 *n* = 39, *N* = 8). **(D)** Phase contrast image of 50 ng/ml PDGF-treated PSC spheroid boundary shows microbead traction toward the spheroid (pink lines, see also Video 1). Black lines on the scatter plot show mean ± SEM. *p < 0.05.

Aiming for a readout of the physiological functions of Piezo1 in PSCs, we investigated whether cellular migration is altered by the Piezo1 activator Yoda1. Indeed, 5 μM Yoda1 stimulate the migration of PSCs seeded on a matrix resembling the desmoplastic matrix found in the PDAC stroma (Figure 3A). Velocity and translocation rise from 0.25 ± 0.01 μm/min and 20.4 ± 2.2 μm (*n* = 83, *N* = 3) under control conditions (0.075% DMSO) to 0.29 ± 0.01 μm/min (*p* = 0.0002) and 41.2 ± 4.3 μm (*p* = 0.0089, *n* = 52, *N* = 3), respectively. The fact that the increase of translocation is more pronounced than that of the velocity indicates a Yoda1-mediated increase in directionality (Figure 3B). Further increasing the concentration of Yoda1 to 15 μM lessens the stimulatory effect on PSC migration (velocity 0.27 ± 0.01 μm/min, *p* = 0.04; translocation 32.5 ± 3.6 μm, *p* = 0.62; *n* = 47, *N* = 3 experiments). In the presence of 50 μM Yoda1, PSCs are no longer accelerated (velocity 0.22 ± 0.01 and translocation 17.0 ± 1.4 μm; *n* = 36, *N* = 3) when compared to equivalent vehicle-treated PSCs (0.25 % DMSO; velocity 0.25 ± 0.02 μm/min, *p* = 0.09; translocation 18.6 ± 1.9 μm, *p* = 0.48; *n* = 45, *N* = 3; Figure 3B).

We next tested whether the Piezo1 activator Yoda1 also affects the migratory behavior of PSCs in a more physiological three-dimensional environment (Figure 3C). To this end we monitored the emigration of PSCs from a spheroid placed in a 3D desmoplastic collagen matrix. The normalized equatorial cross sectional area of PSC spheroids after 24 h increases similarly under control conditions (0.1% DMSO; by 3.5 ± 0.2-fold, *n* = 51 spheroids; *N* = 8) and in the presence of 20 μM Yoda1 (by 3.0 ± 0.2-fold, *n* = 54 spheroids, *N* = 8; *p* = 0.06). However, 20 μM Yoda1 lead to the detachment of more cells from the spheroid (11.0 ± 1.3 cells, *n* = 45 spheroids; *N* = 3) compared to control cells (6.5 ± 0.7 cells, *n* = 40 spheroids; *N* = 3) (Figure 3C).

When monitoring PSC emigration from spheroids with live-cell imaging, it was conspicuous that the surrounding matrix was pulled toward the spheroids. We quantified this phenomenon by tracking the movement of microbeads incorporated into the matrix (Figure 3D). As judged from the bead movement, spheroids pull on the matrix more vigorously in the presence of 20 μM Yoda1 (11.0 ± 0.4 μm/h, *n* = 105 beads, *N* = 3) compared to DMSO-treated spheroids (9.5 ± 0.4 μm/h, *n* = 90 beads, *N* = 3) (Video 1).

### Both Intra- and Extracellular Acidification Impair Piezo1 Activity

**Figure 4:**
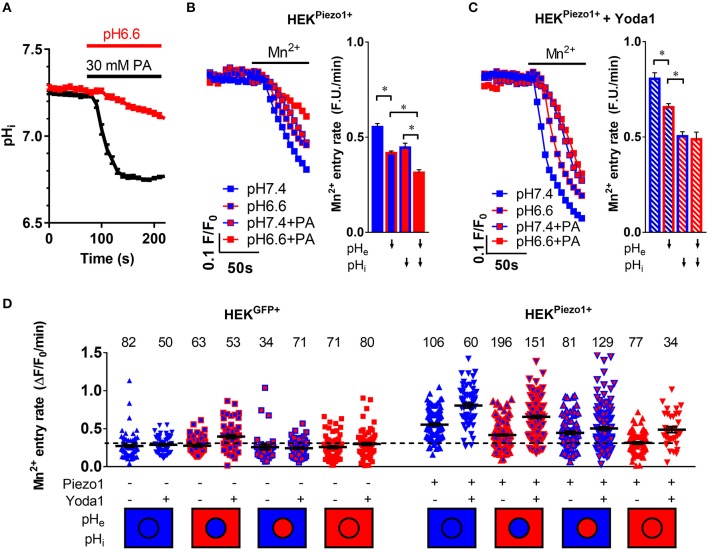
pH-dependent modulation of Piezo1 function. **(A)** Evaluation of intracellular acidification after superfusion of HEK293^Piezo1+^ cells with pH 6.6 Ringer's solution (*n* = 68) or 30 mM propionate (PA; *n* = 64). Error bars represent SEM. **(B,C)** Representative Mn^2+^ quench traces upon superfusion of HEK293^Piezo1+^ cells with solvent **(B)** or 5 μM Yoda1 **(C)** with pH 7.4 or pH 6.6 Ringer's solution, sodium propionate (PA) or pH 6.6 Ringer's solution + propionate (PA). The bar graphs on the right show the statistical evaluation for each condition, where ↓ indicates acidification of the pH_e_ or pH_i_. Column charts show mean ± SEM. *p < 0.05 **(D)** The scatter plot shows Mn^2+^ entry rates after acidification of the intracellular and/or extracellular space in control-transfected or Piezo1-transfected HEK293 cells without or with the co-superfusion with 5 μM Yoda1. The number of evaluated cells is indicated above each group. The dashed line indicates the mean Mn^2+^ entry rate of all control transfected HEK293 cells for better comparability. The intracellular (circle) and extracellular (rectangle) pH environment is color-coded below the groups, with blue meaning physiological pH (pH_i_ = 7.25, pH_e_ = 7.4), whereas red means acidic pH (pH_i_ = 6.7, pH_e_ = 6.6). Black lines show mean ± SEM.

In order to assess how extracellular and/or intracellular acidification affect Piezo1 function, Piezo1-transfected HEK293 cells were treated with pH 6.6 Ringer's solution (*n* = 64, *N* = 3) and/or with 30 mM sodium propionate (*n* = 68, *N* = 3), respectively (Figure 4A). Our pH_i_ measurements indicate that 30 mM sodium propionate acidify HEK293 cells from pH_i_ 7.24 to pH_i_ 6.77 (ΔpH_i_=-0.47±0.01) within 1 min. pH_i_ then reaches a steady state. In contrast, pH_i_ acidification occurs much more slowly upon treatment with pH 6.6 Ringer's solution, as pH_i_ only drops by 0.1 ± 0.002/min. Thus, when assessing Piezo1 function by means of the Mn^2+^ quench technique at *t* = 1 min after the start of the acidification stimulus, we can clearly distinguish between the effect of an intracellular or extracellular acidification.

Acidification of pH_e_ decreases Ca^2+^ influx into Piezo1-transfected HEK293 cells (Figure 4B). The Mn^2+^ quench rate falls from 0.55 ± 0.02 under control conditions (pH_e_7.4) to 0.42 ± 0.01 ΔF/F_0_/min (*n* = 196, *N* = 5; *p* < 0.0001). The acidification of pH_i_ by sodium propionate elicits a similar effect: 0.45 ± 0.02 ΔF/F_0_/min (*n* = 81, *N* = 3; *p* < 0.0001). These effects are additive in Piezo1-transfected HEK293 cells, so that the most pronounced inhibition of Ca^2+^ influx occurs upon dual acidification of the intra- and extracellular space by co-applying pH 6.6 Ringer's solution with 30 mM sodium propionate: 0.31 ± 0.01 ΔF/F_0_/min, *n* = 77, *N* = 3; *p* < 0.0001). This combined acidification diminishes the Mn^2+^ quench to a rate that is only 20% higher than that of control plasmid-transfected HEK293 cells (Figure 4D).

When Piezo1-transfected HEK293 cells are treated with 5 μM Yoda1, pH_e_ acidification (0.66 ± 0.02 ΔF/F_0_/min, *n* = 151, *N* = 4) as well as pH_i_ acidification (0.5 ± 0.02 ΔF/F_0_/min, *n* = 129, *N* = 4) still impair Ca^2+^ influx (Figure 4C). Interestingly, in case of Yoda1 stimulation, intracellular acidification is more effective in inhibiting Ca^2+^ influx than extracellular acidification (*p* < 0.0001), and there appears to be no additional effect of dual acidification (0.48 ± 0.04 ΔF/F_0_/min, *n* = 34, *N* = 2).

### Piezo1 Function in PSCs Is Affected by the Cytoskeleton

**Figure 5:**
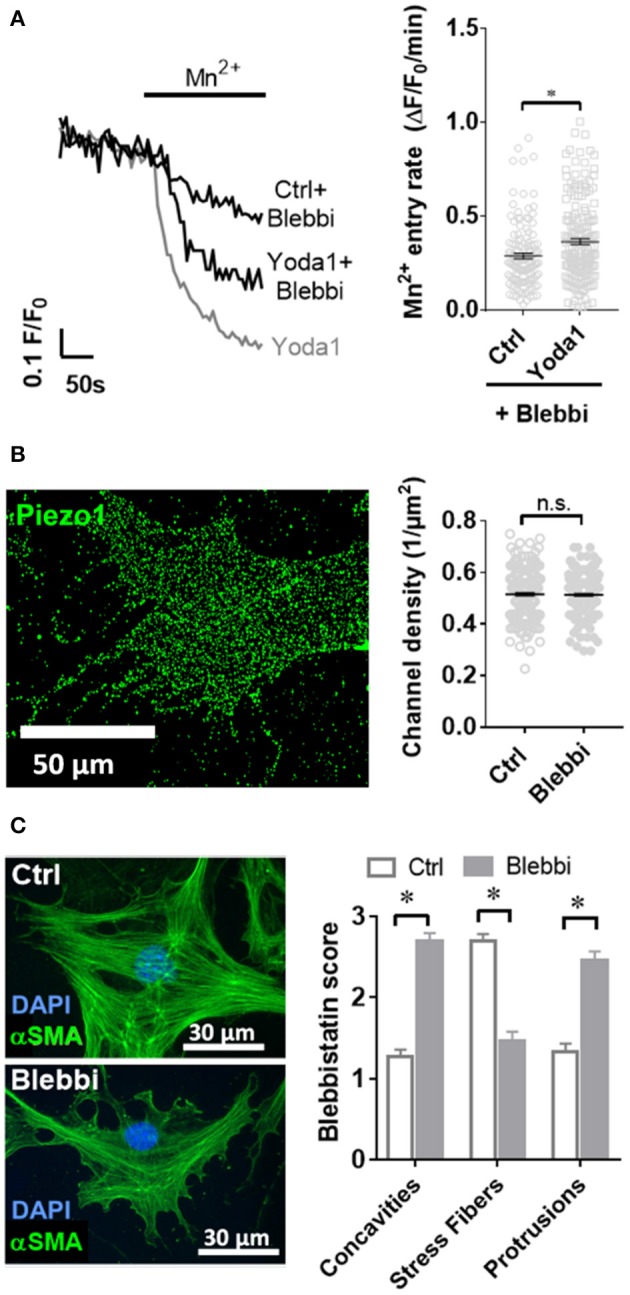
Blebbistatin disrupts the cytoskeleton and impairs Piezo1 function in PSCs. **(A)** Representative Mn^2+^ quench traces of control and 20 μM Yoda1 treated PSCs after pretreatment with 5 μM blebbistatin (+Blebbi) for 15 min. For comparison, a trace of Yoda-1 induced Mn^2+^ quench without blebbistatin pretreatment is shown in gray. The scatter plots show the Mn^2+^ entry rate (*n* = 133 and *n* = 175, respectively, *N* = 3). *p < 0.05. **(B)** Piezo1 immunofluorescence after pretreatment with 5 μM blebbistatin. Scatter plot depicts Piezo1 channel densities (*n* = 300 regions of 30 cells, *N* = 3 experiments). **(C)** Representative immunofluorescence against αSMA (green). Images show PSCs treated with 0.1% DMSO (Ctrl) or blebbistatin (Blebbi). The Blebbistatin score graph depicts the quantification of the cytoskeletal and morphological alterations, detailed in Figure 1. Column charts show mean ± SEM (*n* = 30 cells, *N* = 3).

As it is not known whether Piezo1 is functionally coupled to cytoskeletal tethers in PSCs, we aimed to investigate this by disrupting the actomyosin architecture using the myosin-inhibitor blebbistatin. Upon pre-incubating PSCs with 5 μM blebbistatin for only 15 min, 5 μM of the Piezo1 activator Yoda1 leads to an increase of only 28% in Mn^2+^ quench (0.37 ± 0.02 ΔF/F_0_/min, *n* = 175, *N* = 3) (Figure 5A). This effect is not due to decreased membrane recruitment of the channel. Immunofluorescence staining of Piezo1 in the plasma membrane showed that the channel density after 5 μM blebbistatin treatment (0.47 ± 0.01 channels/μm) does not differ from that of untreated PSCs (*p* = 0.91, Figure 5B). Blebbistatin also causes substantial morphological changes in PSCs, that we evaluated by the criteria shown in Figure 1. Compared to control incubation with 0.1% DMSO (membrane concavities: 1.3 ± 0.1; stress fibers: 2.7 ± 0.1; dendrite-like protrusions: 1.3 ± 0.1; *n* = 30, from *N* = 3), PSCs treated with blebbistatin have more membrane concavities (2.7 ± 0.1) and denrite-like protrusions (2.5 ± 0.1), but less stress fibers (1.5 ± 0.1; *n* = 30, *N* = 3) (Figure 5C).

**Figure 6:**
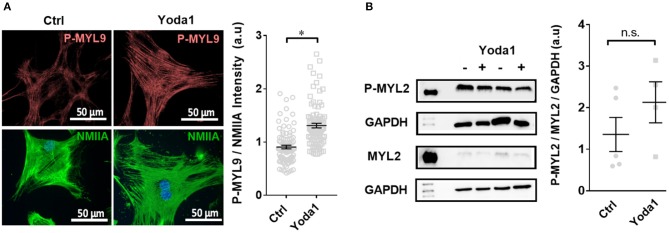
Yoda-1 modifies myosin light chain phosphorylation (P-MYL9) in PSCs. **(A)** Immunocytochemistry images of PSCs treated with vehicle (0.2% DMSO; Ctrl) or 5 μM Yoda1 for 1 h, indicating P-MYL9 (red) and NMIIA (green) of different cells. Scatter plot indicates the total cellular fluorescence intensity of P-MYL9 normalized to NMIIA fluorescence (*n* = 90, *N* = 3). **(B)** Representative Western blots of P-MYL2 and MYL-2, compared to GAPDH, respectively, without or with 5 μM Yoda1 treatment. Scatter plot shows the results for individual PSC lysates (Ctrl: *n* = 5, *N* = 5; Yoda1 *n* = 4, *N* = 4). *p < 0.05.

To elucidate whether Piezo1 modulates cellular contractility, we measured the phosphorylation status of the regulatory myosin light chain MYL9, which is known to have a regulatory role in smooth muscle cells (Figure 6A). We performed immunocytochemistry experiments and normalized the staining of P-MYL9 to that of the non-muscle myosin NMIIA. The treatment with 5 μM Yoda1 increased the ratio of P-MYL9/NMIIA from 0.90 ± 0.03 (vehicle-treated cells) to 1.31 ± 0.04 (*n* = 90, *N* = 3 for both conditions; *p* < 0.0001). For comparison, we also measured the phosphorylation status of MYL2, another regulatory myosin light chain that exerts its main function in cardiac muscle. The Western blot analysis comparing the amount of P-MYL2 to MYL2 in PSC cell lysates shows that the phosphorylation of MYL2 is not significantly different in Yoda1-treated cells (1.4 ± 0.4, *n* = 5, *N* = 5 control lysates vs. 3.2 ± 1.2, *n* = 4, *N* = 4 lysates from Yoda1-treated PSCs; *N* = 5 each; *p* = 0.17) (Figure 6B).

### Acidic pH Disrupts PSC Spheroid Integrity

**Figure 7:**
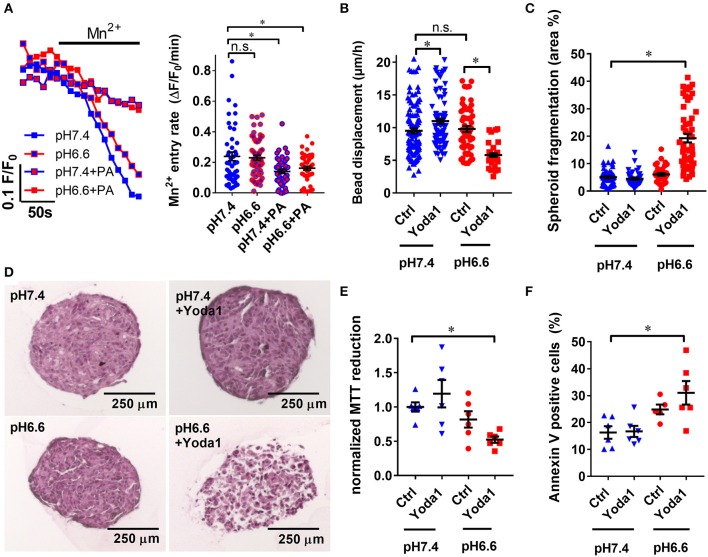
Yoda1 impairs PSC viability in spheroids in acidic conditions. **(A)** Representative Mn^2+^ quench traces of PSCs upon acidification with 30 mM sodium propionate (PA) in pH 6.6 or pH 7.4 Ringer's solution. Scatter plots show statistical evaluation in each condition (*n* = 48, *n* = 57, *n* = 45, *n* = 46, respectively, *N* = 5). **(B)** Microbead traction toward the spheroid in the presence of vehicle (0.1% DMSO, Ctrl) or 20 μM Yoda1 in pH 6.6 or pH 7.4 buffered medium supplemented with 50 ng/ml PDGF (*n* = 48, *n* = 57, *n* = 45, *n* = 46, respectively, *N* = 5). **(C)** Total area of dissociated acellular space within a spheroid section compared to whole cross-sectional spheroid area (*n* = 58, *n* = 42, *n* = 35, *n* = 52 sections, respectively, of spheroids from *N* = 3 mice). **(D)** Representative spheroid sections stained with hematoxylin and eosin. **(E,F)** Scatter plots indicate viability of PSC spheroids in conditions detailed above using MTT reduction assay and Annexin V staining, respectively (*n* = 6, *N* = 3). Data points were normalized to pH 7.4 buffered medium supplemented with 50 ng/ml PDGF and vehicle (0.1% DMSO). *p < 0.05.

To get a better understanding of how PSC physiology is affected in the acidic tumor core, we inspected how changes in intra- and extracellular pH affect Piezo1-elicited responses of PSCs. Our results show that Mn^2+^ entry rate in case of pH_e_ acidification alone (0.23 ± 0.02 ΔF/F_0_/min, *n* = 57, *N* = 5) remains analogous to the control pH 7.4 Ringer's perfusion (0.24 ± 0.02 ΔF/F_0_/min, *n* = 48, *N* = 5; *p* = 0.99) (Figure 7A). On the contrary, intracellular acidification decreases Mn^2+^ entry by 40% (0.14 ± 0.01 ΔF/F_0_/min, *n* = 45, *N* = 5; *p* = 0.003) similarly to combined intra- and extracellular acidification (0.16 ± 0.01 ΔF/F_0_/min, *n* = 46, *N* = 5; *p* = 0.03). Thus, Ca^2+^ influx and thereby Piezo1 function appears to be predominantly inhibited by an intracellular acidification of PSCs.

As PSCs require Ca^2+^ to exert ECM traction, we expected that inhibited Ca^2+^ influx in acidic conditions would impair bead traction in PSC spheroids (Figure 7B). Interestingly, it turned out bead displacement toward PSC spheroids at pH_e_6.6 (9.5 ± 0.4 μm/h, *n* = 57, *N* = 5) is comparable to the displacement at pH_e_7.4 (11.0 ± 0.4 μm/h, *n* = 108, *N* = 5). Even more intriguingly, in the presence of 20 μM Yoda1 at pH_e_6.6, bead displacement is decreased by 40% (5.8 ± 0.3 μm/h, *n* = 37, *N* = 5). To obtain knowledge regarding the mechanism of these phenomena, we investigated the composition of spheroids at different conditions by hematoxylin and eosin staining (Figure 7D). Spheroids treated with 20 μM Yoda1 at pH_e_6.6 are largely fragmented (19.3 ± 1.5 % spheroid cross sectional area, *n* = 52 sections, *N* = 3 spheroids) compared to pH_e_6.6 alone (6.1 ± 0.5 % area, *n* = 35, *N* = 3, *p* < 0.0001) (Figure 7C). In contrast, at pH_e_7.4 there is no difference between vehicle (5.1 ± 0.4 % area, *n* = 58, *N* = 3) and 20 μM Yoda1 treatment (4.5 ± 0.4% area, *n* = 42, *N* = 3, *p* = 0.99). Lastly, we investigated the cellular viability in order to find out whether spheroid fragmentation is due to impaired cell metabolism and viability. MTT reduction normalized to control spheroids cultured at pH_e_7.4 (1.00 ± 0.07, *n* = *n* = 6, *N* = 3) is almost halved at pH_e_6.6 in the presence of 20 μM Yoda1 (0.52 ± 0.05, *n* = 6, *N* = 3, *p* = 0.03) (Figure 7E). For comparison, MTT reduction in the presence of 20 μM Yoda1 at pH_e_7.4 (1.19 ± 0.07, *n* = 6, *N* = 3, *p* = 0.56) and pH_e_6.6 cultured spheroids with vehicle treatment (0.82 ± 0.12, *n* = 6, *N* = 3, *p* = 0.6) is not different. However, loss of MTT reduction can also be attributed to decreased proliferation and decreased cell metabolism and is not a specific readout for cell viability in general. Therefore, we also performed Annexin V staining to detect apoptosis (Figure 7F). Here, cells derived from spheroids that were treated with Yoda1 and pH 6.6 have approximately twice as many Annexin V positive cells (31.0% ± 4.4, *n* = 6, *N* = 3) than under control conditions (16.3% ± 2.3, *n* = 6, *N* = 3, *p* = 0.0046).

## Discussion

Research from recent years has shown that activated myofibroblast-like cells such as pancreatic stellate cells, could serve as potential therapeutic targets in fibrotic conditions. PDAC is characterized by a massive fibrosis produced by PSCs (Robinson et al., 2016) which causes a markedly increased tissue pressure and an acidosis inside the desmoplastic tumor tissue. Our study demonstrates that Piezo1 is a prominent transduction channel involved in adapting the behavior of PSCs to the ambient microenvironmental conditions. Our study focused in particular on the impact of the intra- and extracellular pH for Piezo1 and PSC function.

According to the “force through lipid” model the intrinsically mechanosensitive Piezo1 recognizes changes of tension of the neighboring phospholipid bilayer (Teng et al., 2015). In contrast, the “force through tether” model postulates the necessity of associated proteins e.g., laminin or collagens to translate the force applied to the cell membrane (Lewis and Grandl, 2015; Gaub and Muller, 2017). Our findings support the “force through tether” model, as disruption of the cytoskeleton through blebbistatin inhibited Yoda1-induced activation of Piezo1 (Figure 5A). Upon blebbistatin-mediated inhibition of the myosin ATPase, morphological changes in the cytoskeleton of PSCs are also striking. Similar morphological changes were observed in hepatic stellate cells, who also showed decreased cell contraction in the presence of blebbistatin (Liu et al., 2010). Moreover, blebbistatin-treated PSCs are less sensitive to Yoda1 which then no longer induces Ca^2+^ influx into the cells. A reason for that could have been that the overall Piezo1 density decreases upon blebbistatin treatment. However, our immunocytochemistry data declines this possibility. Thus, our results are consistent with the notion that in PSCs, Piezo1 exhibits its downstream effects through its direct or indirect interaction with the cytoskeleton.

Employing the Piezo1 activator Yoda1, we showed a dose-dependent change in the motility of PSCs plated on a two-dimensional matrix, with enhanced cell migration at lower concentrations and inhibition at high concentrations (Figure 3). In contrast, a recent study focused on transformed fibroblasts (Chubinskiy-Nadezhdin et al., 2019) found that similar concentrations of Yoda1 inhibit collective cell migration in wound healing in a dose-dependent manner. Those experiments investigated directed collective cell migration toward the mechano-chemical gradient in wound healing assay. In our 2D setting we investigated the spontaneous undirected cell migration. Thus, the discrepancies may indicate that Piezo1 has a differential role in the distinct forms of cell migration. Furthermore, we did not see a difference in the overall matrix invasion of the 3D spheroid, which is characterized by a collective and directed cell migration from the spheroid core. However, the number of detached cells increased. In another 3D system investigating lymphatic valve formation, Piezo1 also plays an eminent role in the protrusion of valve leaflets, which is associated with collective cell migration (Nonomura et al., 2018). These findings reinforce our hypothesis that Piezo1 acts differently in different cell migration settings and may direct cells to detach from cell aggregates.

The effects seen by using Yoda1 in cell migration studies can be credited to downstream effects of Piezo1-induced Ca^2+^ influx such as those on the contractile apparatus of PSCs. Ca^2+^ influx leads to phosphorylation of myosin light chains such as MYL9 and activation of the myosin II ATPase. This in turn causes retraction of the trailing edge of cells and mediates the polarity of the leading front edge, which are essential for proper cell migration (Vicente-Manzanares et al., 2009). TRPV4, another mechanosensitive ion channel has also been shown to be involved in the retraction of the trailing part of migrating HEK293 cells (Mrkonjic et al., 2015). Also, cell migration is supported by the concomitant activation of Ca^2+^ activated ion channels such as K_Ca_3.1 which trigger a localized volume loss at the rear part of migrating cells (Schwab et al., 2012).

We showed that Piezo1 activation leads to a boosted phosphorylation state of the regulatory myosin light chain P-MYL9, thus further strengthening the cell cortex (Figure 6). Even though MYL phosphorylation is impacted by Piezo1, MYL and ion channel function are not interchangeable. First, elevation of [Ca^2+^]_i_ through ion channels has many cytoskeleton-independent consequences as reviewed by Gryshchenko et al. (Gryshchenko et al., 2016). Also, interfering with myosins can lead to secondary signaling alterations. This is indicated by the fact that Myo9b^−/−^ dendritic cells have not only impaired migratory capacity but also an altered dendritic cell-T cell interaction (Xu et al., 2014).

We did not observe any changes in total cellular MYL2 phosphorylation in Western blot, which is in line with our work on endothelial cells (Prystopiuk et al., 2018). This phenomenon argues for localized phosphorylation of myosin light chains in the cell cortical area. A beautiful example of such a localized signaling complex has been described in the recent work of Ellefsen et al. where they have shown that traction forces generated by myosin II evoke Piezo1-dependent localized Ca^2+^ flickers (Ellefsen et al., 2019).

Another Ca^2+^ signaling mediator that may be affected after Piezo1-induced Ca^2+^ influx is bradykinin, which is implicated to have key physiological functions in PSCs through the bradykinin receptor 2 (Won et al., 2011; Gryshchenko et al., 2016). This is further supported by the fact that bradykinin in return potentiates Piezo2 currents (Borbiro and Rohacs, 2017). These findings imply that after initial PSC activation by bradykinin, Piezo channel function may be potentiated in PSCs and cause an enhanced Ca^2+^ influx.

Tumor tissue is not only characterized by an increased matrix elasticity, but also by a marked interstitial acidosis. Our experiments showed in a large sample of cells that Piezo1 is modified through extracellular and intracellular acidosis in a partly additive manner (Figures 4B,C). Our observation, that an intracellular acidification with propionate leads to a stronger inhibition of Mn^2+^ quench, is very well in line with the thorough patch-clamp based study of Bae et al., where protonation of Piezo1 was shown to stabilize the channel in an inactivated state (Bae et al., 2015). Another possibility is that the short chain fatty acid propionate directly interacts with the channel and/or modifies the biophysical properties of the cell membrane. As fatty acids have been described to fine-tune the function of Piezo1 (Romero et al., 2019), this may indeed contribute to the effects seen by our propionate treatment. On the other hand, the inhibitory effect of a propionate-induced intracellular acidification might have been underestimated. Exposing cells to 30 mM propionate causes a substantial cell swelling which could increase membrane tension and thereby constitute a trigger for Piezo1 activation.

In prolonged extracellular acidosis, the pH_i_ of stromal cells becomes acidic as well. Under these conditions Piezo1 is likely to not fully function, as demonstrated by our experiments with HEK293 cells (Figures 4B,C) and PSCs (Figure 7A). This indeed seems to have an important protective function for the cells. When acid-primed PSCs are treated with the Piezo1 activator Yoda1, there is less traction applied to the matrix indicated by the decreased bead displacement (Figure 7B). Also, MTT reduction becomes decreased (Figure 7D). Both findings point to a severe loss of spheroid integrity. This loss can be accredited to Ca^2+^ overload and a subsequent induction of cell death which is also supported by the fragmentation of the spheroid (Figure 7C). The decreased viability of Yoda-1 treated acid-stressed spheroids is confirmed by increased Annexin V positivity of cells derived from the spheroid (Figure 7E). Besides apoptosis, a supplementary explanation for the loss of spheroid integrity could be that due to the acidic environment PSCs lose attachment from neighboring cells in a similar manner as we observed previously in melanoma cells (Hofschroer et al., 2017). Along these lines, the fragmentation of the spheroids can be explained by prolonged cell contraction and acidosis-induced loss of cell-to-cell contact.

In conclusion, our study shows that Piezo1 in PSCs is in close cooperation with the cytoskeleton and is sensitive to changes in extra- and intracellular pH. Although the desmoplastic tissue of PDAC exerts a massive mechanical stress onto the cells, the marked tissue acidosis in the tumor core renders Piezo1 less responsive, thus less active in PSCs and cells do not undergo cell death. Because of this protective effect, PSCs may survive to migrate into the well-perfused invasive border of the tumor, where Piezo1 is fully responding to mechanical cues due to the lack of protonation-caused inhibition.

## Data Availability Statement

The datasets generated for this study are available on request to the corresponding author.

## Ethics Statement

The animal study was reviewed and approved by Landesamt für Natur, Umwelt und Verbraucherschutz Nordrhein-Westfalen, permit number 84-02.05.50.15.010.

## Author Contributions

AK was involved in all experiments regarding PSCs, focusing on cell migration, immunofluorescence and Mn^2+^ quench experiments under the guidance of BF, who was involved in interpreting the results and data analysis. OG performed all Mn^2+^ quench and pH measurements with HEK293 cells. SSa created and validated the control plasmid. SSc and IN performed Western blots and analyzed cell migration experiments. AU and MW performed histological analyses of PSC spheroids. AS supervised the project interpreted the data and edited the manuscript with the help of KN. ZP coordinated the project and drafted the manuscript. All authors approved the final version of the manuscript.

### Conflict of Interest

The authors declare that the research was conducted in the absence of any commercial or financial relationships that could be construed as a potential conflict of interest.

## Supplementary Material

Video 1 Extracellular matrix traction by pancreatic stellate cell spheroids.

## Abbreviations

αSMA: Alpha-smooth-muscle actin
ECM: Extracellular matrix
HE: Hematoxylin-eosin stain
PDAC: Pancreatic ductal adenocarcinoma
MYL: Myosin light chain
NMIIA: Non-muscle myosin IIA
P-MYL: Phosphorylated myosin light chain
PDGF: Platelet-derived growth factor
PSC: Pancreatic stellate cell.
